# The establishment of hybrids of the *Daphnia longispina* complex explained by a mathematical model incorporating different overwintering life history strategies

**DOI:** 10.1371/journal.pone.0200802

**Published:** 2018-07-19

**Authors:** Johanna Griebel, Margarete Utz, Joachim Hermisson, Justyna Wolinska

**Affiliations:** 1 Department of Ecosystem Research, Leibniz-Institute of Freshwater Ecology and Inland Fisheries, Berlin, Germany; 2 Department of Biology II, Ludwig Maximilian University Munich, Planegg-Martinsried, Germany; 3 Groningen Institute of Evolutionary Life Science, University of Groningen, Groningen, the Netherlands; 4 Department of Mathematics, University of Vienna, Vienna, Austria; 5 Department of Biology, Chemistry, Pharmacy, Institute of Biology, Freie Universität Berlin, Berlin, Germany; National Taiwan University, TAIWAN

## Abstract

Interspecific hybridization (i.e. mating between species) occurs frequently in animals. Among cyclical parthenogens, hybrids can proliferate and establish through parthenogenetic reproduction, even if their sexual reproduction is impaired. In water fleas of the *Daphnia longispina* species complex, interspecific hybrids hatch from sexually produced dormant eggs. However, fewer hybrid genotypes contribute to the dormant egg bank and their hatching rate from dormant eggs is reduced, compared to eggs resulting from intraspecific crosses. Therefore, *Daphnia* hybrids would benefit from adaptations that increase their survival over winter as parthenogenetic lineages, avoiding the need to re-establish populations after winter from sexually produced dormant eggs. Here, we constructed a mathematical model to examine the conditions that could explain the frequently observed establishment of hybrids in the *D*. *longispina* species complex. Specifically, we compared the outcome of hybrid and parental taxa competition given a reduced contribution of hybrids to hatchlings from the sexually produced dormant egg bank, but their increased ability to survive winter as parthenogenetic lineages. In addition, different growth rates of parental species and differences in average annual temperatures were evaluated for their influence on hybrid production and establishment. Our model shows that increased overwinter performance as parthenogenetic females can compensate for reduced success in sexual reproduction, across all tested scenarios for varying relative growth rates of parental species. This pattern holds true for lower annual temperatures, but at higher temperatures hybrids were less successful. Consequently, hybrids might become less abundant as temperatures rise due to climate change, resulting in reduced diversity and faster differentiation of the parental species.

## Introduction

Interspecific hybridization occurs after secondary contact between partially reproductively isolated species. Its implications for evolutionary and ecological processes have been widely discussed in recent decades [1–4]. Hybrids can display extreme phenotypes due to the combination of genomes of two parental species, and are therefore sometimes able to establish in extreme environments. For example, hybrids of spadefoot toads display longer development times as tadpoles, a strategy beneficial during long dry periods [5], whereas sunflower hybrids are able to establish on sand dunes, desert floors and salt marsh habitats [3]. Interspecific hybrids often suffer reduced abilities to reproduce sexually [6, 7]; however, among cyclical parthenogens, this can be compensated for by increased investment of hybrids into the parthenogenetic part of a reproductive cycle [8, 9].

In the cyclically parthenogenetic water fleas of the *Daphnia longispina* species complex, interspecific hybrids are found worldwide [10–12]. Hybrids are produced during the sexual phase of the *Daphnia* reproductive cycle. In contrast to immediately born parthenogenetic offspring, sexual offspring hatch after diapause, from dormant eggs (ephippia). This is how *Daphnia* survive unfavourable conditions, for example winter. Fewer hybrid genotypes are present in the dormant egg bank and their hatching rate is lower than for offspring resulting from intraspecific crosses [8, 13]. As evident from the rarity of backcrosses and F2-hybrid generations in natural *Daphnia* communities [9, 13, 14], F1-hybrids are also rather unsuccessful in further sexual reproduction [8]. However, F1-hybrids can successfully compete with their parental species during the parthenogenetic part of the *Daphnia* life cycle [15–17]. They may even survive winter without going through diapause; i.e. as parthenogenetic lineages [9]. In our recent experimental study, *Daphnia galeata × D*. *longispina* F1-hybrids originating from several shallow lakes (i.e. strongly influenced by harsh winters) had an increased rate of survival as parthenogenetic lineages under simulated winter conditions (4 °C, low food and 8:16 hours light-dark photoperiod [18]). The ability to overwinter as parthenogenetic lineages can play an important role during recolonization of the water body in spring: individuals that are present first in the water column might quickly dominate the entire community, inhibiting the establishment of genotypes hatching from dormant eggs later in the season [19–21]. However, the trade-off between reduced success in surviving winter as sexually produced dormant eggs, and increased ability to survive as an active population, has not been evaluated previously in terms of prospects for hybrid establishment.

We constructed a mathematical model simulating *Daphnia* community dynamics between two parental species, F1- and F2-hybrids, and their species-specific backcrosses, to determine which biological features and/or which environmental conditions explain the frequently observed dominance of F1-hybrids [10–12]. We evaluated the reduced probability of hybrid genotypes to contribute to sexually produced dormant egg bank, but their increased ability to overwinter as parthenogenetic lineages, using parameter values derived from published work [8, 13]. Here, we adjusted the seasonal function for F1-hybrids during winter and used the survival data from our previous experimental study [18]; seasonal changes in temperature and light, parameters strongly affecting *Daphnia* growth during the year, were simulated by applying a sinusoidal function from [22]. Finally, we tested how establishment of F1-hybrids is influenced by variation in relative growth rates of the parental species (phenomenon known to be driven by ecologically realistic conditions, [23–26]) and by differences in average annual temperatures.

## Model

Individuals were divided into six classes: two parental species (*j* = 1 and *j* = 2), F1-hybrids (*j* = 3), backcrosses to respective parental species (*j* = 4 and *j* = 5) and F2-hybrids (*j* = 6). For each class the numbers of asexual individuals (*A*), sexual individuals (*S*) and dormant eggs, ephippia (*E*), were modelled using the following differential equations:

$$\frac{dA_{j}}{dt}=r_{j}\;\sigma (t)\;A_{j}(t)(1-\frac{\sum_{i=1}^{6}{(A}_{i}(t)+S_{i}(t))}{\sigma (t)K})-s_{j}(t)A_{j}(t)+{2\;h}_{j}{(t)\;E}_{j}(t)$$ (1)

$$\frac{dS_{j}}{dt}=s_{j}(t)A_{j}(t)-m\;S_{j}(t)$$ (2)

$$\frac{dE_{j}}{dt}=({1-e}_{j})f\varphi_{j}(t)-h_{j}{(t)\;E}_{j}(t)$$ (3)

**Fig 1:**
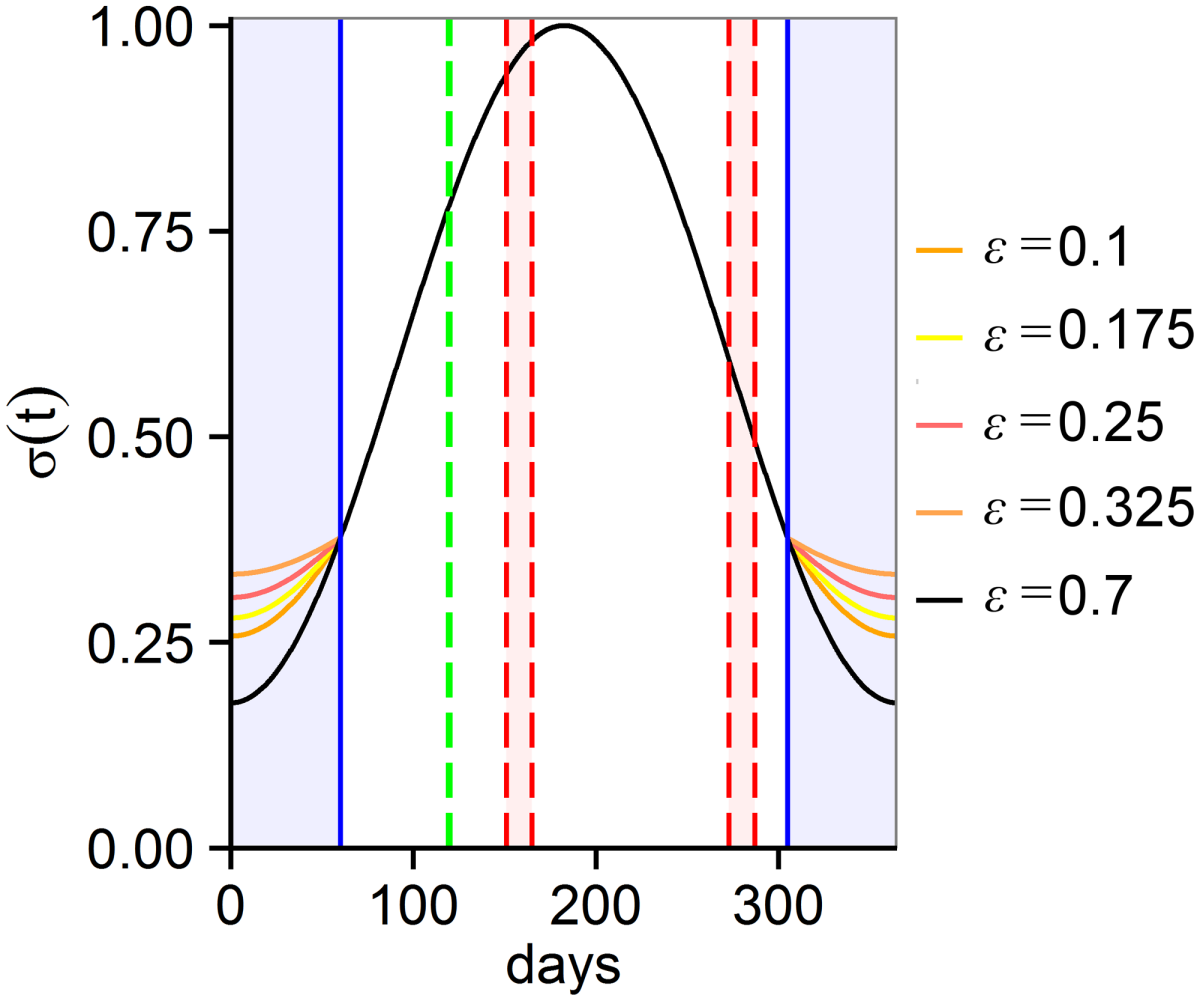
Adjustment of seasonal forces in *Daphnia* by *σ(t)*. The black line represents the standard curve for *ε* = 0.7 [22], while the orange lines (*ε* = 0.1, 0.175, 0.25, 0.325) were used to obtain higher growth rates of F1-hybrids during winter (120 days from October to February). Blue vertical lines mark the winter period during which *σ(t)* differs for F1-hybrids. The red lines indicate the periods of sexual reproduction in early spring and autumn (May and September). The green line shows the hatching period from ephippia in spring (April).

The number of sexual encounters was multiplied by *f* (number of ephippia produced per day) and by the fraction of ephippia containing eggs (*1-e*_*j*_, Eq (3)), as unsuccessful mating would result in empty ephippia, *e*_*j*_. The growth of asexual individuals is logistic with a specific intrinsic growth rate *r*_*j*_ for each class. The carrying capacity *K* is shared by all six classes, as well as asexual and sexual individuals. During two periods of the year (two weeks in May and September, days: 151–165 and 273–287, [13]) a fraction *s*_*j*_*(t)* of asexual individuals switches to sexual reproduction. Sexual individuals die at a fixed mortality rate *m*. At the beginning of April (days 119–120), ephippia (containing two embryos) hatch at a rate *h*_*j*_*(t)* and result in asexual individuals, mimicking a photoperiod-driven hatching of dormant eggs [27]. Seasonal forces (seasonal cycle of light and temperature) were included by multiplying the carrying capacity *K* (affected by resource availability, changing with light conditions) and the growth rate *r*_*j*_ (affected by metabolic rates, changing with temperature) by a periodic function of time *t* (Fig 1) with *ε* set to 0.7 as proposed by [22]:

$$\sigma (t)=\frac{1-\varepsilon \;\text{cos}\left(\frac{2\pi t}{365}\right)}{1+\varepsilon }$$ (4)

The production of ephippia was calculated using the harmonic mean developed by [28], which accounts for the fact that both females and males have to be present. The model was set up with equal fractions of males and sexual females for the different classes:

$$\varphi (t)=\frac{2\;k\;\frac{S_{i}}{2}(t)\;\frac{S_{l}}{2}(t)}{\sum_{j}\left\{\frac{S_{j}}{2}\right\}\;}$$ (5)

**Fig 2:**
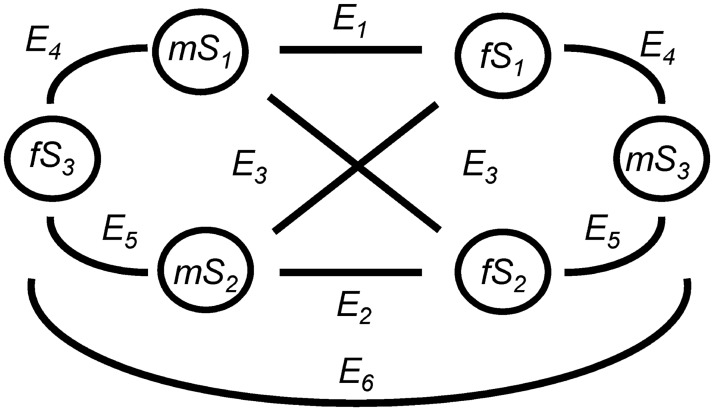
Possible encounters within and between the two parental species (class 1 and 2) and F1-hybrids (class 3) during the phase of sexual reproduction, resulting in ephippia of parental species, F1-hybrids, both backcrosses and F2-hybrids (*E*_*j*_, *j* = 1–6). *m*: fraction of males, *f*: fraction of sexual females. *S*_*1*_*-S*_*3*_: sexual individuals of class 1–3.

Here, *k* is the number of ephippia produced per sexual encounter, which was set to one. *S*_*i*_*(t)* and *S*_*l*_*(t)* are the number of sexual individuals of class *i and l* (here *i*, *l* = class *1–3*) at time *t*. For simplification, sexual individuals of the backcrosses and F2-hybrids (classes *j* = 4, 5, 6, see above) do not take part in sexual reproduction. Ephippia containing the F1-hybrid (or backcross) class are produced by encounters of sexual females of one parental species with males of the other parental species, and vice versa (or by encounters of F1-hybrid females with males of any parental species, and vice versa) (Fig 2). To simulate deviation from random mating, the sexual encounters were divided into individuals that only mate with individuals from their own class (fraction *c*) and individuals that mate randomly with any other individual (*1-c*). The fraction *c* was set the same for all three classes (no empirical data exist for this parameter). These lead to the following equations:

$${\;\varphi }_{j(t)}=\frac{\frac{1}{2}\;c\;S_{j}{(t)\;c\;S}_{j}(t)}{c\;S_{j}(t)}+\frac{\frac{1}{2}\;(1-c)S_{j}{(t)\;(1-c)S}_{j}(t)}{\left(1-c)\;{\;(S}_{1}(t)+S_{2}(t){+S}_{3}(t)\right)}==\;\frac{\;S_{j}(t)\;(S_{j}(t)+c({{\;S}_{1}(t)+S}_{2}(t)+S_{3}(t)-S_{j}(t)))}{2\;(S_{1}(t)+S_{2}(t)+S_{3}(t))}\;for\;j=1,2$$ (6)

$$\varphi_{3(t)}=\frac{\;{(1-c)\;S}_{1}{(t)\;(1-c)S}_{2}(t)}{{(1-c)\;(S}_{1}(t)+S_{2}(t){+S}_{3}(t))}=\frac{\;(1-c)\;S_{1}(t)\;S_{2}(t)}{{\;S}_{1}(t)+S_{2}(t){+S}_{3}(t)}$$ (7)

$$\varphi_{4(t)}=\frac{\;{(1-c)\;S}_{1}{(t)\;(1-c)S}_{3}(t)}{{(1-c)\;(S}_{1}(t)+S_{2}(t){+S}_{3}(t))}=\frac{\;(1-c)\;S_{1}(t)\;S_{3}(t)}{{\;S}_{1}(t)+S_{2}(t){+S}_{3}(t)}$$ (8)

$$\varphi_{5(t)}=\frac{\;{(1-c)\;S}_{2}{(t)\;(1-c)S}_{3}(t)}{{(1-c)\;(S}_{1}(t)+S_{2}(t){+S}_{3}(t))}=\frac{\;(1-c)\;S_{2}(t)\;S_{3}(t)}{{\;S}_{1}(t)+S_{2}(t){+S}_{3}(t)}$$ (9)

$$\varphi_{6(t)}=\frac{\frac{1}{2}\;c\;S_{3}{(t)\;c\;S}_{3}(t)}{c\;S_{3}(t)}+\frac{\frac{1}{2}\;(1-c)S_{3}{(t)\;(1-c)S}_{3}(t)}{\left(1-c)\;{\;(S}_{1}(t)+S_{2}(t){+S}_{3}(t)\right)}=\frac{\;S_{3}(t)\;(S_{3}(t)+c(S_{1}(t)+S_{2}(t)))}{2\;(S_{1}(t)+(S_{2}(t)+S_{3}(t))}$$ (10)

Several scenarios were evaluated by solving the differential Eqs (1)–(3) numerically using Mathematica 10.3 [29]. Unless otherwise stated, parameter values are as described in Table 1. Starting densities were set to 0.001 individuals per litre for each parental species and zero individuals for the other classes. Carrying capacity was set to 150 individuals per litre [18].

**Table 1:** Parameters of the model. The phrase “empty ephippia” refers to ephippia not containing any eggs.

parameter	value	unit	Description	Reference
*m*	0.15	day^-1^	death rate	[30]
*K*	150	no. L^-1^	carrying capacity	[18]
*s*_*j*_	0.5	day^-1^	fraction of individuals of class *j* switching to sexual reproduction	[13]
*f*	0.14	day^-1^	number of ephippia produced per day	[31]
*e*_*1*,*2*_	0.7		fraction of empty ephippia of class *j = 1*,*2*	[13]
*e*_*3*_	0.8		fraction of empty ephippia of class *j = 3*	[13]
*e*_*4*,*5*_	0.75		fraction of empty ephippia of class *j = 4*,*5*	[13]
*e*_*6*_	0.85		fraction of empty ephippia of class *j = 6*	[13]
*c*	0.75		fraction of sexual individuals mating within own class	[8]
*h*_*1*,*2*_	0.1	day^-1^	hatching rate from ephippia of class *j = 1*,*2*	[8]
*h*_*3*,*4*,*5*,*6*_	0.05	day^-1^	hatching rate from ephippia of class *j = 3*,*4*,*5*,*6*	[8]

### Lower contribution of hybrids to hatchlings from the dormant egg bank, but their increased overwinter performance as asexual females

A 100-year time span has been chosen for simulations, as F1-hybrids’ increased overwinter survival as asexual females has been observed in a man-made lake of similar age [18]. Hatching rate *h*_*j*_ was set 50% lower and the number of empty ephippia *e*_*j*_ was set about 15% higher in hybrids compared to parental species [8, 13]. The fraction of sexual individuals that mate within their own class was set at 75% (*c* = 0.75). Growth rates were set the same for parental species, F1-hybrids and backcrosses (*r*_*j*_ = 0.35 for *j* ≠ 6). The growth rate for F2-hybrids was set lower, assuming a hybrid breakdown (*r*_*6*_ = 0.3, scenario A). Unfortunately, no data on fitness of F2-hybrids exist, as too few F2-hybrids are found in natural communities [9, 12] to be successfully established in laboratory cultures. We tested to what extent the better overwintering success of F1-hybrids as asexual lineages changes their establishment success. Therefore, the *ε* of the seasonal force function (4) was set lower than in the original function (*ε* = 0.1, 0.175, 0.25 and 0.325) to increase the growth rate of F1-hybrids (*r*_*3*_) during winter (120 days; November till February, Fig 1). These changes in *ε* result in 50 to 80% higher growth rates of F1-hybrids at the minimum of the seasonal function (at day 365). In laboratory experiments, growth rates during winter conditions (4 °C, low food and short photoperiod) were found to be seven times higher for F1-hybrids compared to parental species [18]. We opted for more conservative values to exclude laboratory artefacts (e.g. experimental clones were sampled in spring, when only successful survivors were present). Additionally, the growth rate of F1-hybrids (*r*_*3*_) was varied for different calculations (range: 0.30 to 0.35), to estimate whether increased winter performance may compensate for a generally lower fitness of asexual individuals in F1-hybrids. For example, hybrids in the laboratory had lower growth rates compared to parental species when raised at 20 °C [24].

### Different growth rate scenarios for the parental species

In addition to scenario (A) in which both parental species have the same growth rates (which is unlikely in nature), we evaluated four other scenarios for successful F1-hybrid establishment;

B) one parental species has a higher intrinsic growth rate than the other [24]; here: *r*_*1*_ = 0.35 and *r*_*2*_ = 0.3;

C) the intrinsic growth rates of the parental species alternate every second year (for example, because of changing parasite pressure, [26]); here: *r*_*1*_ = 0.3 then 0.35 and *r*_*2*_ = 0.35 then 0.3;

D) the intrinsic growth rates of the parental species change within a year (because species might have reverse fitness under different seasonal conditions, [25]); here: switch at day 166, *r*_*1*_ = 0.3 then 0.35 and *r*_*2*_ = 0.35 then 0.3;

E) the second parental species enters with a higher intrinsic growth rate, after the system has been dominated by a single parental species for 100 years (for example, due to eutrophication, [23]); here: *r*_*1*_ = 0.3 and *r*_*2*_ = 0.35;

The growth rates of backcrosses (*r*_*4*_ and *r*_*5*_) were set to the average of the respective parental species and F1-hybrids. The growth rate of F2-hybrids was set lower (*r*_*6*_ = 0.3), whereas the growth rate of F1-hybrids (*r*_*3*_) was varied for different calculations (range: 0.30 to 0.35).

### Different average annual temperatures

For all scenarios (A)–(E), the effect of decreased/increased average annual temperature (by *x* °C) on the establishment of hybrids was tested by multiplying the growth rate of Eq (1) by the parameter:

$$q=2^{\frac{x}{10}}$$ (11)

The formula is derived from the temperature coefficient Q_10_, which measures the change of biological processes induced by a temperature increase of 10 °C. The coefficient Q_10_ has been evaluated for *Daphnia* [32] and applied in zooplankton-phytoplankton models [33, 34]. Similarly to [33, 34], the following values of decreased/increased average annual temperature were tested: *x* = -5 °C / -3 °C / +3 °C / +5 °C.

## Results

### Lower contribution of hybrids to hatchlings from the dormant egg bank, but their increased overwinter performance as asexual females

**Fig 3:**
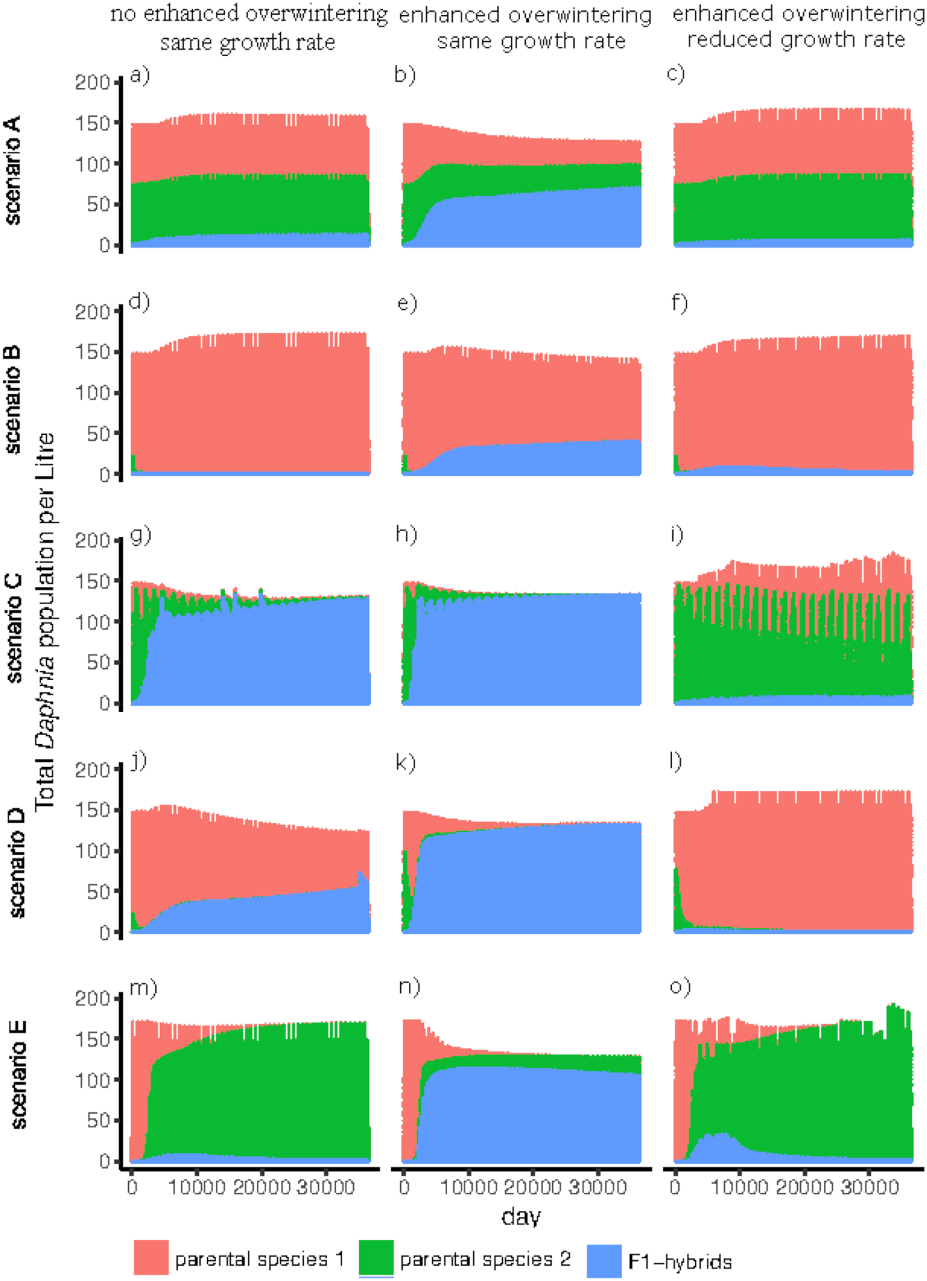
Total numbers of individuals (asexual and sexual) of the two parental species and F1-hybrids over 36500 days (100 years) with: F1-hybrids having reduced hatching success from sexually produced dormant eggs (50% lower hatching rates and about 15% higher fraction of empty ephippia, compared to parental species), no increased/enhanced overwinter performance for F1-hybrids *(left column*), increased overwinter performance of F1-hybrids (ε = 0.1 in φ(t) for an 80% higher growth rate during winter, *middle column*), and F1-hybrids having increased overwinter performance and reduced growth rate during the year (*right column*). The *top row* represents scenario A, where parental species have the same growth rate, the *mid top row* represents scenario B, where parental species 1 has a higher growth rate than parental species 2, the *mid row* represents scenario C, where the absolute growth rate of parental species alternates every two years, the *mid bottom row* represents scenario D, where growth rate of parental species alternates within the year and the *bottom row* represents scenario E, where the second species was introduced after 100 years, having a higher growth rate (graphs show the 100 years after parental species 2 had entered the system).

Analysis of the model showed that under a scenario of non-random mating and reduced hatching success from sexually produced dormant eggs for F1-hybrids (i.e. 50% lower hatching rate (*h*_*3*_ = 0.05) and about 15% more empty ephippia (*e*_*3*_ = 0.8) compared to parental species (*h*_*1*,*2*_ = 0.1 and *e*_*1*,*2*_ = 0.7)), F1-hybrids were present at low numbers (< 20 *Daphnia* / L, proportion of 13%, Fig 3a). However, in the case of an increased overwinter performance of F1-hybrids as asexual females, the number of established F1-hybrids increased under all simulated differences in the seasonal function (4) (i.e. 50%, 60%, 70% and 80% higher growth rates of F1-hybrids during winter compared to parental species, S1 Fig). Backcrosses as well as F2-hybrids were present in low numbers (S2 Fig). After an increase of the F1-hybrids’ growth rate to 80% during winter (*ε* = 0.475 in Eq 4), F1-hybrids reached abundances of more than 50% in the *Daphnia* community (S1 Fig). Therefore, the seasonal function with ε = 0.1 leading to 80% higher growth rate during winter was applied for F1-hybrids. If the general growth rate of hybrids was reduced by about 15% (r_3_ = 0.31) over the whole year, F1-hybrids were still detected, but below five *Daphnia* individuals per litre. They then coexisted with the parental species but were not able to dominate the system (Fig 3c) as they otherwise did when their growth rate was equal to the growth rate of the parental species (Fig 3b). Backcrosses and F2-hybrids were no longer present (S3c Fig).

### Different growth rate scenarios for the parental species

If F1-hybrids experienced reduced hatching success from sexually produced dormant eggs, they could not establish in two of the four further tested scenarios (i.e. additional to scenario A): in scenario B (where one parental species had a lower growth rate than the other parental species, Fig 3d) and scenario E (where the second species with a higher growth rate was introduced after 100 years). In scenario E, F1-hybrids occurred for about 20 years but then vanished (Fig 3m). When the growth rates of the parental species alternated, either between (scenario C) or within years (scenario D), F1-hybrids were detectable after ten years and quickly dominated the system (proportions of 60–75%, Fig 3g and 3j). However, the smaller the difference in the average growth rates was between F1-hybrids and parental species in these scenarios (C, D), the smaller the numbers of hybrids (data not shown). In the case of parental species having altered growth rates within the year (scenario D), the parental species with a higher growth rate in the second half of the year was more abundant in the system than the parental species that had a higher growth rate at the beginning of the year (Fig 3j).

If the overwinter performance of F1-hybrids was increased (seasonal function with ε = 0.1 for 160 days: from November till February), then F1-hybrids occurred in all four scenarios of different growth rates for the parental species (B–E, Fig 3). In scenario B (where one parental species had a lower growth rate than the other parental species, Fig 3e) F1-hybrids coexisted with the parental species, while in the other scenarios they dominated the system. In scenario B, the growth rate of F1-hybrids could only be reduced by 0.9% (*r*_*3*_ = 0.347) over the whole year while still facilitating their abundance, compared to 12% in scenario A (Fig 3c). In scenarios C and D (growth rates of the parental species alternating between or within years, respectively), F1-hybrids achieved dominance more rapidly (proportions of 85% after 5 years) as is the case when overwinter performance was not increased via the seasonal function (Fig 3h and 3k). F1-hybrids were even able to coexist with the parental species when their growth rate was reduced by as much as 13% (*r*_*3*_ = 0.31) and 9.5% (*r*_*3*_ = 0.32), respectively (Fig 3i and 3l). In scenario E (where the second species with a higher growth rate was introduced after 100 years), increased overwinter performance of F1-hybrids facilitated their appearance shortly after the addition of the second parental species, and led to their dominance after about 20 years (66%, Fig 3n, the graph shows the 100 years after the new parental species was introduced). Furthermore, successful establishment of F1-hybrids was facilitated if their growth rates were reduced by up to 3% (*r*_*3*_ = 0.34). However, time until establishment was longer in such a case and the abundance of F1-hybrids decreased after 50 years to low numbers (Fig 3o). In all scenarios, backcrosses and F2-hybrids occurred at low numbers (below ten individuals per litre) when overwinter performance of F1-hybrids was increased (S3 Fig).

### Different average annual temperatures

**Fig 4:**
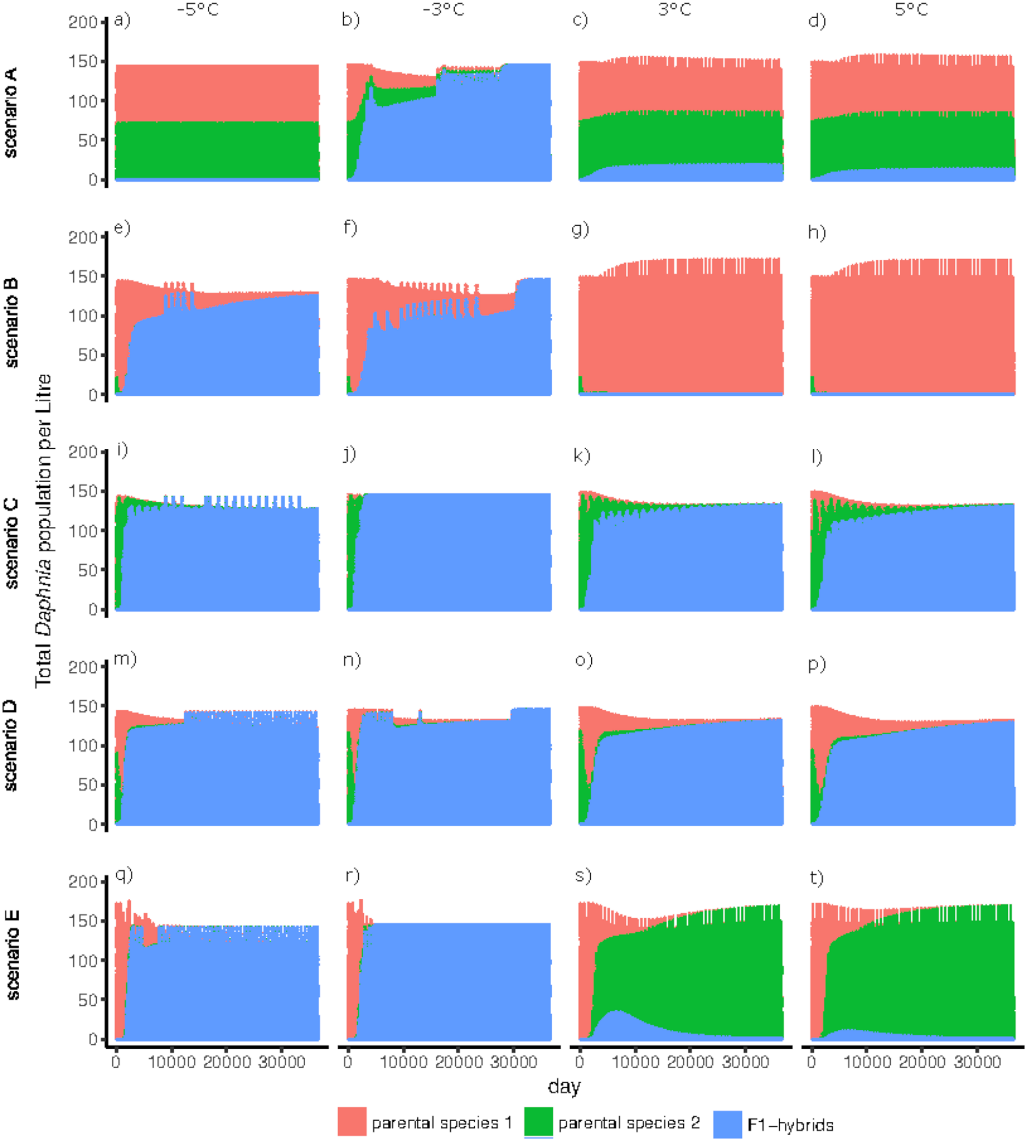
Total numbers of individuals (asexual and sexual) of the two parental species and F1-hybrids over 36500 days (100 years) with F1-hybrids having reduced hatching success from sexually produced dormant eggs (50% lower hatching rates, and about 15% higher fraction of empty ephippia, compared to parental species) and parental species having different growth rates. The *ε* of the seasonal function of F1-hybrids differed during winter to simulate their increased/enhanced overwinter performance as asexual individuals (ε = 0.1 for an 80% higher growth rate). The growth rate of F1-hybrids is 0.35. Differences in the average annual temperature (changes of -3 °C, -5 °C, +3 °C, and +5 °C) were applied for each scenario of different parental growth rates. The *top row* represents scenario A, where parental species have the same growth rate, the *mid top row* represents scenario B, where parental species 1 has a higher growth rate than parental species 2, the *mid row* represents scenario C, where the absolute growth rate of parental species alternates every two years, the *mid bottom row* represents scenario D, where growth rate of parental species alternates within the year and the *bottom row* represents scenario E, where the second species was introduced after 100 years, having a higher growth rate (graphs show the 100 years after parental species 2 had entered the system).

Under lower average annual temperatures (differences of -3 °C and -5 °C), F1-hybrids established as fast or faster compared to the previous analyses with *x* = 0 °C (Fig 4). The only exception was a temperature difference of -5 °C in scenario A, where F1-hybrids did not establish (Fig 4a). In addition, the number of F2-hybrids was higher, whereas the number of backcrosses was lower, than in the analysis with *x* = 0 °C (S4 Fig). Higher average annual temperatures (differences of +3 °C and +5 °C) resulted in the failure of hybrids to establish in scenarios A, B and E (Fig 4). The numbers of F2-hybrids and backcrosses were lower, or these classes did not exist at all, when temperatures increased (+3 °C and +5 °C, S4 Fig).

## Discussion

Lower contribution to hatchlings from the dormant egg bank is a clear disadvantage for *Daphnia* hybrids. Our model indicates that F1-hybrids sometimes do not establish or establish only at low numbers. Studies on reproductive barriers in *Daphnia* have shown that time differences in production of sexual stages [35], as well as occupation of different habitats within a lake by the two parental species [36], both reduce encounter probability, and thus the possibility to hybridize. Consequently, fewer F1-hybrid dormant eggs are produced than if mating was random, and the viability of these eggs is anyway reduced [8]. However, if only F1-hybrids have increased overwintering performance as parthenogenetic females, they are not only able to become established, but can dominate the system.

The results of our model can explain patterns of *Daphnia* hybrid occurrence observed in nature. For example, although F1-hybrids were shown to have reduced success in surviving winter as dormant eggs, in some years they dominated the entire *Daphnia* community of lake Greifensee (Switzerland) [8, 13]. F1-hybrids first occurred in that lake in the 1950s, when the level of phosphorus rose and the second parental species *D*. *galeata* invaded the lake [23]. This is consistent with scenario E in our model, where the introduction of a second species resulted in the quick establishment of F1-hybrids, with their increased overwinter performance as asexual females. Across several shallow lakes (Germany), F1-hybrids occurred together with only one parental species [9, 10]. This confirms that hybrids do not need to be produced newly every season; otherwise both parental species would need to be present to allow hybrid persistence. Once created, hybrids can be maintained in the population through asexual reproduction, including surviving winter as parthenogenetic lineages. Indeed, F1-hybrids collected from these lakes had a higher overwintering survival probability than lineages of parental species, as demonstrated experimentally [18]. Moreover, genotype data of the aforementioned communities also suggest that F1-hybrids survive winter mainly as parthenogenetic females [9]. Our model predicts coexistence or dominance of F1-hybrids as long as the overwinter performance of F1-hybrids as parthenogenetic females is increased, even if only one parental species is present in the long-term. Interestingly, only low numbers of F2-hybrids and backcrosses are usually present in natural habitats [8–10], again consistent with the results of the model.

In our model, average annual temperatures of lakes had an important effect on the occurrence of F1-hybrids. Colder temperatures result in faster establishment and dominance of F1-hybrids, because only very low numbers of parental species survive as asexual females. Consequently, F1-hybrids reach high abundances before the parental species hatch from ephippia. Surviving as asexual females can lead to a competitive advantage in spring, when surviving *Daphnia* can quickly reproduce parthenogenetically, and their offspring are born during an algal bloom [21], resulting in later dominance (i.e. priority effects, [37]). Interestingly, in scenario A no F1-hybrids were detected when the average annual temperature was reduced by 5 °C, suggesting that below a certain temperature, no asexual females survive over winter. In this case, hatchlings from ephippia become the main colonization source in spring, resulting in a disadvantage to hybrids. Hybrids are also at a disadvantage at the opposite end of the temperature range, though for a different reason: high temperatures lead to large numbers of parental species surviving as asexual females, therefore hybrids lose their priority advantage after the winter and cannot establish. The model thus predicts that hybrids thrive at an intermediate temperature range (cold, but not too cold). Indeed, we have observed previously that a successful F1-hybrid clone (with experimentally proven increased overwinter survival as asexual females) was replaced in its natural habitat by the parental species after a warm winter [18].

One limitation of this study is that our model assumes that F2-hybrids have single low values for asexual reproductive rate (*r*_*6*_) and hatching rate (*h*_*6*_), and a single high ratio of empty ephippia (*e*_*6*_). This simplifies the real situations because F2 hybrids (and backcrosses) can potentially show high variation in fitness between individuals [38, 39] and theoretically produce very fit lineages. However, across several dozen lakes sampled multiple times (work of our group and those of Petrusek, Schwenk and Spaak–ca. 20 published papers), such highly fit (i.e. highly abundant) F2- or backcross lineages have not been observed. This is in contrast to, for example, sometimes highly abundant F1- lineages [18]. Then, our model assumes that a constant fraction (c = 0.75) of individuals in species 1, species 2 and F1-hybrid class mate with individuals of the same class. Unfortunately, no data exist on that parameter, and this is why we set similar value for all three classes, to reduce potential bias.

Overall, our model shows that hybrids’ increased overwinter performance as asexual females might explain their frequently observed establishment and dominance in natural *D*. *longispina* communities. Even if F1-hybrids contribute little to hatchlings from the dormant egg bank, their increased overwinter performance through asexual reproduction can compensate for that. As the strength of hybrids’ increased overwinter performance depends on average annual temperatures, and temperatures of lakes are rising throughout the world [40] [41], the number of parental species surviving winter will likely increase. Consequently, F1-hybrids may become less abundant, leading to faster genetic differentiation of the parental species.

## Supporting information

S1 Fig Total numbers of individuals (asexual and sexual) of the two parental species and F1-hybrids over 36500 days (100 years), with F1-hybrids having reduced success in hatching success from sexually produced dormant eggs (50% lower hatching rates and about 15% higher fraction of empty ephippia, compared to parental species). Parental species and F1-hybrids have the same growth rates during the year (*r*_*1*,*2*,*3*_ = 0.35). The *ε* of the seasonal function of F1-hybrids differed during winter to simulate increased/enhanced overwinter performance of F1-hybrids as asexual females, compared to parental species: a) ε = 0.325 (50% higher growth rate, b) ε = 0.25 (60% higher growth rate), c) ε = 0.175 (70% higher growth rate), d) ε = 0.1 (80% higher growth rate).
(EPS)

S2 Fig Total numbers of individuals (asexual and sexual) of the two backcrosses and F2-hybrids over 36500 days (100 years), with F1-hybrids having reduced hatching success from sexually produced dormant eggs (50% lower hatching rates and about 15% higher fraction of empty ephippia, compared to parental species). Parental species and F1-hybrids have the same growth rates during the year (*r*_*1*,*2*,*3*_ = 0.35). The *ε* of the seasonal function of F1-hybrids differed during winter to simulate increased/enhanced overwinter performance of hybrids as asexual females, compared to parental species: a) ε = 0.325 (50% higher growth rate, b) ε = 0.25 (60% higher growth rate), c) ε = 0.175 (70% higher growth rate), d) ε = 0.1 (80% higher growth rate).
(EPS)

S3 Fig Total numbers of individuals (asexual and sexual) of the two backcrosses and F2-hybrids over 36500 days (100 years) with: F1-hybrids having reduced hatching success from sexually produced dormant eggs (50% lower hatching rates and about 15% higher fraction of empty ephippia, compared to parental species), no increased/enhanced overwinter performance of F1-hybrids as asexual females *(left column*), increased overwinter performance of F1-hybrids (ε = 0.1 for a 80% higher growth rate during winter, *middle column*), and F1-hybrids having increased overwinter performance and reduced growth rate during the year (*right column*). The *top row* represents scenario A, where parental species have the same growth rate, the *mid top row* represents scenario B, where parental species 1 has a higher growth rate than parental species 2, the *mid row* represents scenario C, where the absolute growth rate of parental species alternates every two years, the *mid bottom row* represents scenario D, where growth rate of parental species alternates within the year and the *bottom row* represents scenario E, where the second species was introduced after 100 years, having a higher growth rate (graphs show the 100 years after parental species 2 had entered the system).
(EPS)

S4 Fig Total numbers of individuals (asexual and sexual) of the two backcrosses and F2-hybrids over 36500 days (100 years) with F1-hybrids having reduced hatching success from sexually produced dormant eggs (50% lower hatching rates and about 15% higher fraction of empty ephippia, compared to parental) and parental species differing in growth rates. The *ε* of the seasonal function of F1-hybrids differed during winter to simulate increased/enhanced overwinter performance of asexual individuals, compared to parental species (ε = 0.1 for an 80% higher growth rate). The growth rate of F1-hybrids and backcrosses is 0.35, while F2-hybrids have a growth rate of 0.3. Differences in the average annual temperature (changes -3 °C, -5 °C, +3 °C, +5 °C) were applied for each different scenario of different parental growth rates. The *top row* represents scenario A, where parental species have the same growth rate, the *mid top row* represents scenario B, where parental species 1 has a higher growth rate than parental species 2, the *mid row* represents scenario C, where the absolute growth rate of parental species alternates every two years, the *mid bottom row* represents scenario D, where growth rate of parental species alternates within the year and the *bottom row* represents scenario E, where the second species was introduced after 100 years, having a higher growth rate (graphs show the 100 years after parental species 2 had entered the system).
(EPS)
